# P-selectin (CD62P) and soluble TREM-like transcript-1 (sTLT-1) are associated with coronary artery disease: a case control study

**DOI:** 10.1186/s12872-020-01663-2

**Published:** 2020-08-24

**Authors:** Li Shen, Tianlun Yang, Ke Xia, Zhiqiang Yan, Juanjuan Tan, Lei Li, Yingchun Qin, Wei Shi

**Affiliations:** 1 Department of Cardiology, The Third Hospital of Changsha, Changsha, Hunan China; 2 Department of Cardiology, Xiangya Hospital, Central South University, Changsha, Hunan China; 3 Central Laboratory, Fengxian Hospital Affiliated to the Southern Medical University, Shanghai, China; 4 Joint Research Center for Precision Medicine, Shanghai Jiao Tong University Affiliated Sixth People’s Hospital South Campus, Shanghai, China; 5 The Third School of Clinical Medicine, Southern Medical University, Guangzhou, Guangdong China; 6 Shanghai University of Traditional Chinese Medicine, Shanghai, China

**Keywords:** Coronary artery disease, Acute myocardial infarction, CD62P, platelet P-selectin ligand, Soluble TREM-like transcript-1, Acute coronary syndromes

## Abstract

**Background:**

Platelet activation plays a crucial role in the pathogenesis of coronary artery disease (CAD). Platelet P-selectin (CD62P) is a classic platelet activation indicator on the platelet surface, and soluble TREM-like transcript-1 (sTLT-1) is a new indicator. However, the relationship between these two markers and CAD, especially in acute coronary syndrome (ACS), has not been elucidated. This study aimed to investigate CD62P expression on the platelet surface and sTLT-1 expression in serum, as well as to assess their relationship with CAD.

**Methods:**

We measured the levels of CD62P and sTLT-1 in 83 patients with CAD compared to 49 controls. The association of these indicators with age, blood pressure, lipid profile, body mass index, and liver injury marker level were also examined.

**Results:**

CD62P concentration was higher in CAD patients than in the control group (*P* < 0.01), especially in acute myocardial infarction (AMI) patients (*P* < 0.01). Serum sTLT-1 concentration was higher in the AMI and unstable angina pectoris (UAP) groups than in the normal control (NC) group (*P* < 0.01).

**Conclusions:**

The consistency of sTLT-1 and CD62P expression levels in CAD patients indicates that sTLT-1 level, the same as CD62P, may be a new marker of platelet activation that is positively related to CAD.

## Background

As coronary artery disease (CAD) continues to present an increasing global burden, early detection and timely management of risk factors are crucial to reduce morbidity and mortality. Platelet activation plays a crucial role in the pathogenesis of CAD [1]. Circulating activated platelets are thought to trigger ischemic complications after angiography, angioplasty, and vascular surgery. The onset of CAD, especially in acute coronary syndrome (ACS), is closely associated with enhanced platelet adhesion and aggregation. Therefore, early detection and antiplatelet therapy are critically important for the treatment of coronary heart disease.

P-selectin (CD62P) is a type of glycoprotein stored in the Weibel-Palade bodies of vascular endothelial cells and platelet α-granules. Activated CD62P is a transmembrane protein expressed on the platelet surface [2]. During platelet activation, the expression of CD62P on the surface of the cell membrane is dramatically increased, and is accompanied by a simultaneous increase in the expression level of plasma soluble CD62, which plays an important role in the initiation, formation, and expansion of the thrombus. Furthermore, CD62P exposure is known to be an established marker for platelet activation. Current research has found that the percentage expression of CD62P on platelets without any stimulation by platelet-activating factors, such as ADP, collagen, and thrombin, is higher in CAD patients compared to participants in a control group [3]. Tenaglia et al. [4] also reported significantly greater CD62P staining on atherectomy specimens from patients with unstable angina than from patients with stable angina. These findings support the concept that increased CD62P expression has important repercussions in atherosclerosis, and therefore, targeting this pathway may be a therapeutic option.

TREM-like transcript-1 (TLT-1), which coexists with CD62P in platelet alpha particles, is a recently discovered platelet activation indicator expressed on the platelet membrane surface [5, 6]. When a soluble TLT-1 (sTLT-1) is released in the plasma, it enhances platelet aggregation [2, 6], which can also reflect the expression of platelet membrane TLT-1 [7]. Despite the proof of the biological functions CD62P plays in platelets and sTLT-1 plays in the serum, there is little information about their role in CAD. Therefore, this study was performed to elucidate the association between CD62P, sTLT-1, and CAD. Based on the potential link between CD62P, sTLT-1, and platelet activation, we systematically analyzed the correlation of these indicators with CAD.

## Methods

### Clinical samples

This study enrolled consecutive patients with CAD who had coronary angiographic stenosis greater than 50% (*n* = 83). Among these patients, 29 had acute myocardial infarction (AMI), 26 had unstable angina pectoris (UAP), and 28 had stable angina pectoris (SAP). Patients were compared with 49 healthy control subjects from the physical examination center without CAD. Patients with any evidence of tumor or systemic disease were excluded. The patients agreed to participate in this study and signed the informed consent form. A detailed clinical history was collected from all of the patients, including clinical characteristics.

### Information collection

Clinical data of the general condition (age, gender, height, and weight), medical history (hypertension, diabetes, or stroke), personal history (smoking or drinking), medication history, family history, and laboratory tests were obtained from all patients.

### Measurement of plasma

Blood venous samples were collected directly in plastic tubes in the morning. The collected blood samples were about 5 ml, and were placed in a vacuum blood collection vessel, centrifuged for 3 min (1500 r/min) to separate the upper platelet-rich plasma, and placed in a sterile Epoxy tube. The expression level of CD62P on the platelet surface was detected by flow cytometry (BD FACSCalibur, USA). The concentration of sTLT-1 in the serum was measured by enzyme-linked immunosorbent assay, which was performed according to the manufacturer’s instructions.

### Statistical analysis

The statistical analysis was performed by IBM SPSS version 22.0 software. Quantitative data are presented as mean ± standard deviation (SD). Any differences among ≥ 3 groups were evaluated by ANOVA with Scheffe’s test for parametric variables and by the Kruskal-Wallis test with the Steel-Dwass test for nonparametric variables. Any differences between two groups were evaluated with an unpaired t-test for parametric variables. Correlations between sTLT-1, CD62P, and the severity of coronary atherosclerosis were evaluated by Spearman’s rank correlation test. A *P*-value of <0.05 was considered to indicate statistical significance.

## Results

### Clinical characteristics of the participants

The details of demographic, clinical, and laboratory data of the 132 study participants are listed in Table 1. The results showed that there were no significant differences in age, sex, body mass index (BMI), systolic blood pressure (SBP), diastolic blood pressure (DBP), or platelet count between the CAD and NC groups. Low density lipoprotein (LDL) and triglyceride (TG) levels in the ACS group were higher than those in the NC group (^△^*P* < 0.05). A stepwise decrease in high density lipoprotein (HDL) levels was found in the AMI and UAP groups that did not appear in the control group (^△^*P* < 0.05). The blood glucose (BG) level in the AMI group was higher than in the NC group (^★^*P* = 0.03).

**Table 1:** Clinical characteristics (Mean ± SD)

	NC (*n* = 49)	SAP (*n* = 28)	UAP (*n* = 29)	AMI (*n* = 26)
Age (years)	55.10 ± 9.19	56.89 ± 8.59	57.28 ± 8.69	58.62 ± 7.52
Men/female (n)	(27/22)	(15/13)	(16/13)	(15/11)
BMI (KG/m^2^)	23.24 ± 3.06	24.11 ± 1.28	24.33 ± 1.96	24.19 ± 2.43
TG (mmol/L)	1.12 ± 0.53	1.21 ± 0.59	1.52 ± 0.76^△^	1.76 ± 1.06^△^
LDL (mmol/L)	2.24 ± 0.55	2.38 ± 0.65	2.81 ± 0.43^△#^	2.99 ± 0.43^△#^
HDL (mmol/L)	1.43 ± 0.44	1.32 ± 0.32	1.14 ± 0.23^△#^	1.10 ± 0.26^△#^
BS (mmol/L)	5.04 ± 0.53	5.3 ± 0.53	5.37 ± 0.96	5.72 ± 1.52^★^
ALT (IU/L)	28.58 ± 7.01	26.08 ± 6.05	40.23 ± 15.38	43.12 ± 19.83
SBP (mmHg)	125.8 ± 20.02	130.5 ± 19.24	131.4 ± 22.35	129.3 ± 19.03
DBP (mmHg)	79.08 ± 8.05	80.32 ± 8.59	76.65 ± 11.28	77.38 ± 12.66
Platelet count (10^9^/L)	193.1 ± 44.47	191.2 ± 46.55	185.93 ± 45.24	187.12 ± 42.48

*Abbreviations*: *BMI* Body mass index, *TG* Total triglycerides, *LDL-C* Low-density lipoprotein cholesterol, *HDL-C* High-density lipoprotein cholesterol, *Glu* Fasting glucose, *BS* Blood glucose, *ALT* Alanine aminotransferase, *SBP* Systolic blood pressure, *DBP* diastolic blood pressure; Data are presented as mean values ± standard deviation or proportions (%). *P*-value represents the difference among the four groups: vs. NC: ^△^*P* < 0.05; vs. SAP: ^#^*P* < 0.05; vs., NC: ^★^*P* = 0.03

### Levels of plasma sTLT-1 and platelet CD62P in patients with CAD vs. healthy controls

**Fig. 1:**
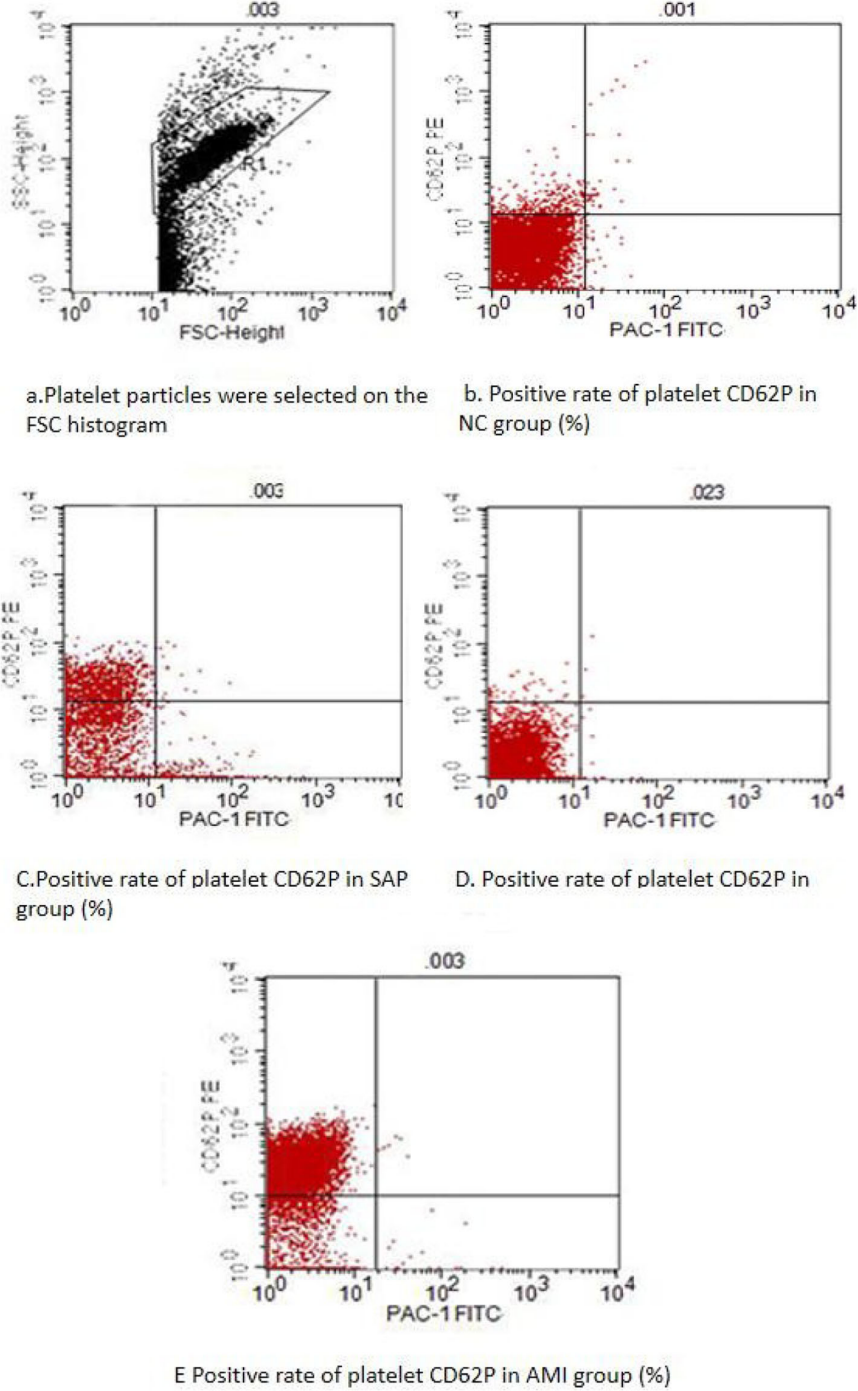
A region (R1) is drawn around the platelets, which were identified according to their characteristic size (**a**); The CD62P positivity rate of platelets in the NC, SAP, UAP, and AMI groups (%) (**b**-**e**)

**Fig. 2:**
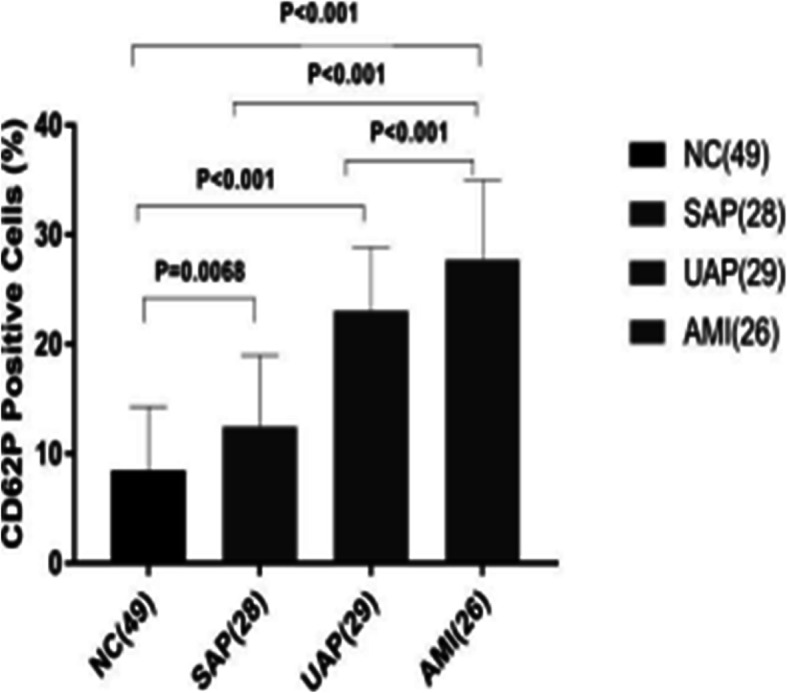
Comparison of the positivity rate of CD62P in the platelet membrane in each group (NC group, SAP group, UAP group, and AMI group): The CD62P levels were significantly higher in CAD participants. NC vs. SAP: *P* = 0.0068; vs. UAP: *P* < 0.001; vs. AMI: *P* < 0.001; especially in AMI: vs. SAP: *P* < 0.001; vs. UAP: *P* < 0.001.△*P* < 0.05; vs. UAP: #*P* = 0.011; vs SAP: ★*P* < 0.01

We found that the expression level of CD62P in the platelet membrane of the CAD group was significantly higher than that expressed in the NC group, and the difference was statistically significant (Figs. 1, 2 and Table 2). The expression level of platelet CD62P in the AMI group was significantly higher than in the SAP group (*P* = 0.011), and the difference was statistically significant (*P* < 0.01).

**Fig. 3:**
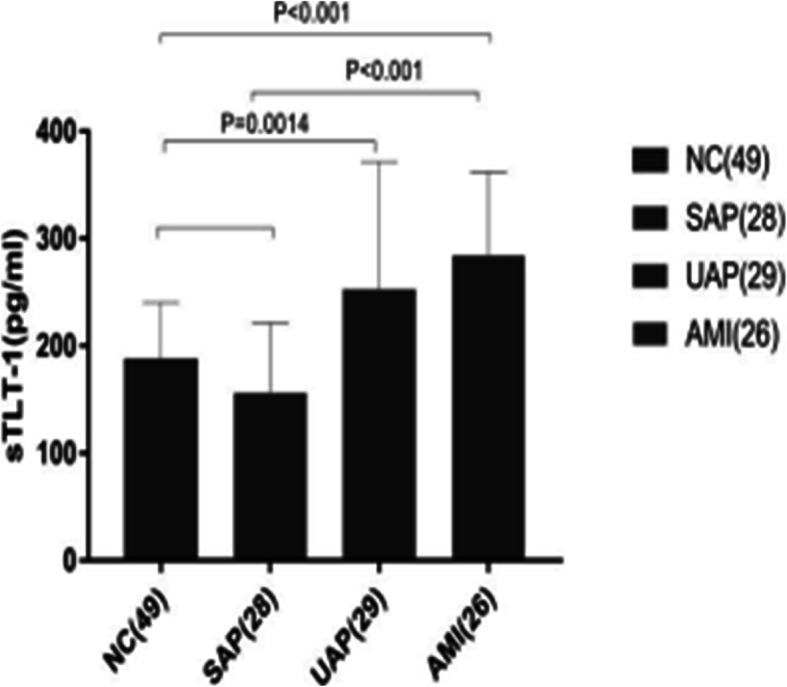
Serum sTLT-1 level compared among the four groups (NC group, SAP group, UAP group, and AMI group). The sTLT-1 levels were significantly higher in CAD participants. NC vs. SAP: *P* = 0.0235; vs. UAP: *P* = 0.0014; vs. AMI: *P* < 0.001; especially in AMI: vs.: SAP: *P* < 0.001

**Table 2:** Levels of plasma sTLT-1 and platelet CD62P in patients with CAD vs. healthy controls

	NC (*n* = 49)	SAP (*n* = 28)	UAP (*n* = 29)	AMI (*n* = 26)
CD62P Positive Cells (%)	8.44 ± 5.83	12.46 ± 6.63^△^	23.02 ± 5.83^△^	27.2 ± 7.3^△#*^
sTLT-1 (pg/ml)	187.62 ± 52.61	156.21 ± 64.85	252.63 ± 118.93^△^	284.35 ± 77.56^△*^

*Abbreviations*: *CD62P* Platelet-selectin, *sTLT-1* Soluble TREM-like transcript-1; Data are presented as mean values ± standard or proportions (%). *P*-value represents the difference among the four groups: vs NC: ^Δ^ *P* < 0.05; vs SAP: ^#^ *P* < 0.05; vs UAP: ^*^P < 0.05

Given that elevated plasma levels of sTLT-1 are implicated in the activation of platelets, we measured plasma sTLT-1 levels in patients diagnosed with CAD. Soluble TLT-1 levels were significantly increased in patients with AMI and UAP compared with matched, healthy control participants (Fig. 3 and Table 2), and a significant correlation was observed between patients with AMI versus those with SAP (*P* < 0.01).

#### Correlation analysis of serum sTLT-1, platelet membrane CD62P, and other risk factors for coronary heart disease

Spearman regression was used to analyze the correlation between platelet membrane CD62P and serum sTLT-1 and the other risk factors of coronary heart disease in ACS patients, including HDL, LDL, and TG (Tables 3 and 4). The results showed that the serum sTLT-1 level was positively correlated with TG (*r* = 0.42), LDL (*r* = 0.17), and CD62P (*r* = 0.49), and negatively correlated with HDL (*r* = − 0.28). Among these factors, there was a positive correlation of sTLT-1 with BS (*r* = 0.2), CHO (*r* = 0.47), LDL (*r* = 0.32), body weight (*r* = 0.21), and sTLT-1 (*r* = 0.49), but there was a negative correlation of sTLT-1 with HDL (*r* = − 0.29).

**Table 3:** Correlation analysis of serum sTLT-1 and other CAD risk factors

	sTLT-1	
	r	*P*-value
BS	0.18	0.07
TG	0.42	0.00
LDL	0.17	0.05
HDL	−0.28	0.00
Pt	−0.077	0.34
Age	0.15	0.09
Weight	0.26	0.77
BMI	0.33	0.71
Sex	−0.15	0.14
CD62P	0.49	0.00

*r* Spearman’s correlation coefficients

**Table 4:** Correlation analysis of the platelet membrane CD62P and other CAD risk factors

	CD62P	
	r	*P*-value
BS	0.25	0.04
TG	0.38	0.65
LDL	0.32	0.00
HDL	−0.29	0.00
Pt	−0.27	0.61
Age	0.13	0.15
Weight	0.21	0.02
BMI	0.29	0.04
Sex	0.12	0.22
sTLT-1	0.49	0.00

*r* Spearman’s correlation coefficients

## Discussion

The World Health Organization divides coronary heart disease into the following five clinical types: asymptomatic, angina pectoris, myocardial infarction, ischemic cardiomyopathy, and sudden death. ACS is referred to as coronary atherosclerosis plaque rupture and thrombosis, or vasospasm and clinical syndrome. Acute or subacute myocardial ischemia includes UAP and AMI. The typical presentation of myocardial ischemia is chest pain, and its development is associated with platelet activation. The adhesion molecule CD62P plays a dominant role in modulating the interactions between platelets and the endothelium, and is involved in acute cardiovascular events [8]. Studies have confirmed that CD62P is one of the classic indicators of platelet activation. The activation of platelets is a promising target for antiplatelet therapy.

The results of our study showed that the platelet CD62P level was significantly higher in the AMI, UAP, and SAP groups than in the normal control group, and furthermore, the platelet CD62P level in the AMI group was significantly higher than that in the UAP and SAP groups, which was consistent with previous research results. The results of this experiment showed that there was no significant difference between the UAP group and the SAP group, which may be related to the small number of specimens collected in this experiment.

TLT-1 is a type-1 transmembrane protein [5, 6, 9] that is highly and exclusively expressed in megakaryocytes and platelets [10], stored in α-granules and possibly in another platelet compartment, and is rapidly upregulated on the surface of activated platelets, with CD62P showing the most obvious increase in expression [11]. Upon platelet activation, TLT-1 is transported to the membrane, where it enhances Ca2+ influx and promotes platelet aggregation. TLT-1 is a new player in platelet aggregation that has been proven to have a significantly higher sTLT-1 expression level in patients with chest pain than in the control group [12, 13]. In acute respiratory distress syndrome, the results showed that sTLT-1 expression level was closely related to coronary heart disease, and it was also one of the indexes of platelet activation. In a murine model of acute lung injury, Morales-Ort’ız et al. showed that TLT-1 mediates fibrinogen deposition in the lungs and facilitates platelet-neutrophil release during transmigration [14]. In mice, inhibition of TLT-1 has been shown to reduce thrombosis in carotid artery thrombosis models and to protect mice from excessive bleeding during pulmonary embolism [12]. Studies have proven that the serum level of sTLT-1 is correlated with the incidence of ACS and has a certain predictive value [15]. However, the specific mechanism needs to be studied further.

The results of our experiment showed that the serum sTLT-1 level in the AMI group and the UAP group was significantly higher than in the NC group and the SAP group. The expression level of sTLT-1 in the AMI group was significantly higher than in the SAP group. Therefore, it is speculated that in the early stage of rapid platelet activation, TLT-1 may also show a similar increase in expression to CD62P, a platelet activation indicator.

Additionally, the level of serum sTLT-1 in ACS patients can provide some reference for the diagnosis of coronary heart disease. Antiplatelet therapy is the cornerstone of treatment for thrombotic diseases, especially ACS. This study explored the function of platelets in CAD. TLT-1 is a newly discovered indicator of platelet activation. Further study and better understanding of this indicator may provide new directions and targets for the diagnosis of thrombosis-related diseases and anti-platelet therapy.

## Conclusion

The consistency of sTLT-1 and CD62P expression levels in CAD indicates that sTLT-1 level may be a new marker of platelet activation that is positively related to CAD, especially in ACS patients. The serum TLT-1 level was significantly higher in patients with CAD, and especially those with ACS, than it was in the normal population, suggesting that TLT-1 may be involved in the disease occurrence of patients with ACS.

## Supplementary information

**Additional file 1.**

## Data Availability

The datasets used and/or analyzed during the current study are available from the corresponding author upon reasonable request.

## Abbreviations

CAD: Coronary artery disease
ACS: Acute coronary syndrome
sTLT-1: Soluble TREM-like transcript-1
AMI: Acute myocardial infarction
UAP: Unstable angina pectoris
SAP: Stable angina pectoris
BMI: Body mass index
SBP: Systolic blood pressure
DBP: Diastolic blood pressure
LDL: Low density lipoprotein
HDL: High density lipoprotein
TG: Triglyceride
LDL-C: Low-density lipoprotein cholesterol
HDL-C: High-density lipoprotein cholesterol
Glu: Fasting glucose
BG: Blood glucose
ALT: Alanine aminotransferase
NC: Normal control
